# 17β-Estradiol promotes sex-specific dysfunction in isolated human arterioles

**DOI:** 10.1152/ajpheart.00708.2022

**Published:** 2023-01-06

**Authors:** Gopika SenthilKumar, Boran Katunaric, Henry Bordas-Murphy, Micaela Young, Erin L. Doren, Mary E. Schulz, Michael E. Widlansky, Julie K. Freed

**Affiliations:** ^1^Department of Physiology, Medical College of Wisconsin, Milwaukee, Wisconsin, United States; ^2^Cardiovascular Center, Medical College of Wisconsin, Milwaukee, Wisconsin, United States; ^3^Department of Anesthesiology, Medical College of Wisconsin, Milwaukee, Wisconsin, United States; ^4^Department of Medicine, Medical College of Wisconsin, Milwaukee, Wisconsin, United States; ^5^Department of Surgery, Medical College of Wisconsin, Milwaukee, Wisconsin, United States; ^6^Department of Pharmacology, Medical College of Wisconsin, Milwaukee, Wisconsin, United States

**Keywords:** endothelium, estrogen, flow-induced dilation, hormone therapy, microcirculation

## Abstract

Despite data showing that estrogen is vasculoprotective in large conduit arteries, hormone therapy (HT) during menopause has not proven to mitigate cardiovascular disease (CVD) risk. Estrogen exposure through prolonged oral contraceptive use and gender-affirming therapy can also increase cis- and trans-females’ risk for future CVD, respectively. The microvasculature is a unique vascular bed that when dysfunctional can independently predict future adverse cardiac events; however, studies on the influence of estrogen on human microvessels are limited. Here, we show that isolated human arterioles from females across the life span maintain nitric oxide (NO)-mediated dilation to flow, whereas chronic (16–20 h) exposure to exogenous (100 nM) 17β-estradiol promotes microvascular endothelial dysfunction in vessels from adult females of <40 and ≥40 yr of age. The damaging effect of estrogen was more dramatic in arterioles from biological males, as they exhibited both endothelial and smooth muscle dysfunction. Furthermore, females of <40 yr have greater endothelial expression of estrogen receptor-β (ER-β) and G protein-coupled estrogen receptor (GPER) compared with females of ≥40 yr and males. Estrogen receptor-α (ER-α), the prominent receptor associated with protective effects of estrogen, was identified within the adventitia as opposed to the endothelium across all groups. To our knowledge, this is the first study to report the detrimental effects of estrogen on the human microvasculature and highlights differences in estrogen receptor expression.

**NEW & NOTEWORTHY** Microvascular dysfunction is an independent predictor of adverse cardiac events; however, the effect of estrogen on the human microcirculation represents a critical knowledge gap. To our knowledge, this is the first study to report sex-specific detrimental effects of chronic estrogen on human microvascular reactivity. These findings may offer insight into the increased CVD risk associated with estrogen use in both cis- and trans-females.

## Graphical abstract

**Figure:**
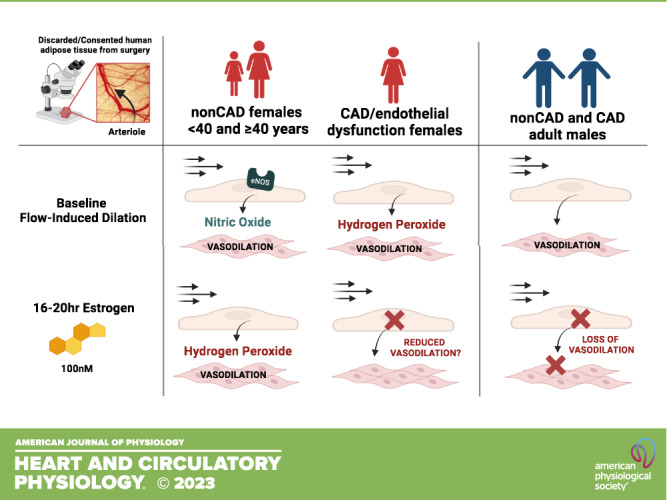

## INTRODUCTION

Cardiovascular disease (CVD) risk in females increases during low-estrogen states such as menopause (1). Although estrogen has been shown to increase endothelial nitric oxide (NO) production (2, 3) and improve large artery function in both cis- and trans-females (4, 5), these beneficial effects do not translate to improved CVD outcomes with estrogen therapy for unknown reasons. In fact, CVD risk can increase in cis-females taking combined oral contraceptives (6) and postmenopausal hormone therapy (HT) (7), as well as in trans-females undergoing gender-affirming therapy (8).

Most studies that have examined the influence of estrogen on vascular function have focused on large, conduit arteries. However, microvascular dysfunction is a more powerful predictor of major adverse cardiac events (MACEs) (9) and key differences exist between conduit and resistance arterioles. For instance, conduit arteries predominantly rely on endothelial NO to dilate in response to flow [flow-induced dilation (FID)], therefore a decrease in NO bioavailability results in reduced vasodilatory capacity. On the contrary, resistance arterioles have the ability to compensate for the loss of NO by transitioning to hydrogen peroxide (H_2_O_2_) as the primary mediator of dilation (10). The anti-inflammatory properties of NO drastically differ from the proinflammatory nature of H_2_O_2_, which can have a profound impact on the surrounding parenchyma.

Studies that have assessed human microvascular health across the female life span and in response to estrogen therapy (e.g., high/chronic estrogen states) are lacking and is the primary aim of this study. The influence of exogenous 17β-estradiol (E_2_) on the vasoactive mediator produced during shear was examined in both female- and male-isolated human arterioles. In addition to microvascular reactivity studies, estrogen receptor expression [ER-α, ER-β, and G protein-coupled estrogen receptor (GPER)] in human arterioles was also examined.

## METHODS

### Tissue Collection

Otherwise discarded, fresh surgical adipose tissue (peritoneal, visceral, subcutaneous) was obtained and placed in ice-cold HEPES buffer. Deidentified patient information was collected via the REDCap database at the Medical College of Wisconsin. Patients scheduled for surgeries that likely produce discard tissue were consented before surgery. Patients under the age of 80 yr and 0–1 risk factor(s) for coronary artery disease [CAD; hypertension, active smoker, hyperlipidemia, congestive heart failure, and diabetes mellitus (type 1 or 2)] were classified as non-CAD. Patients were classified as CAD if they had a confirmed diagnosis of obstructive atherosclerosis via cardiac catheterization. If isolated arterioles from patients with ≥2 risk factors dilated primarily to H_2_O_2_ before any treatment (control), they were classified as having endothelial dysfunction (ED). Tissue from patients with a positive COVID-19 test result in the past year were excluded from the study. The protocols used were approved by the Medical College of Wisconsin’s Institutional Review Board.

### Microvascular Function Studies

Peripheral arterioles from non-CAD patients (0–1 risk factors for CAD), patients formally diagnosed with CAD, and those classified as having ED were dissected and incubated in EBM-2 media (10% fetal bovine serum) with 17-β estradiol (16–20 h, 100 nM, Sigma-Aldrich E8875) or vehicle (100% ethanol 0.1% vol/vol; 16–20 h). Resistance vessels (maximal internal vessel diameter 162 ± 63 µm; means ± SD) were cannulated on glass micropipettes of matched resistance and placed in a pH and temperature (37°C)-controlled organ chamber with Kreb’s buffer. The arterioles were equilibrated for 30 min at 30 mmHg, followed by 30 min at 60 mmHg. Arterioles were then preconstricted to 30%–70% of their passive internal diameters using 0.2–2 nM endothelin-1 (ET-1). Changes in internal diameters were measured using videomicroscopy at steady state (5 min) following graded increases in flow by adjusting the pressure gradient (5–100 cmH_2_O) of the reservoirs in equal and opposite directions as originally described by Kuo et al. (11). To determine the vasoactive mediator required for FID, *N*^ω^-nitro-l-arginine methyl ester (l-NAME; 10^−4^ mol/L), an NO synthase inhibitor, or polyethylene glycol‐catalase (PEG‐catalase; 500 U/mL), an enzyme that promotes catabolism of H_2_O_2_, was added to the organ chamber for 30 min before the initiation of flow. An endothelium-independent vasodilator, papaverine, (10^−4^ mol/L), was administered following each flow-response experiment to determine the vessel’s maximal diameter. Only two experiments were performed on each arteriole; between experiments, vessels were washed 3× with Krebs buffer and equilibriated at 60 mmHg for 30 min. Vessels that exhibited an inability to hold tone for 10 min following endothelin-1 preconstriction were excluded. The percent change in vessel diameter was calculated at each flow rate, with 0% representing the ET-preconstricted diameter and 100% representing the maximal diameter of the vessel.

Our laboratory has functional historical control data dating back to 2006. All studies since 2006 have been conducted using the same experimental techniques, similar equipment that undergoes routine calibration, and exclusion criteria based on ET-1 response. All technicians received similar training on proper techniques in which to conduct these studies. We used these historical control data from 2010 to 2019 to discern differences in FID, as well as smooth muscle function of arterioles based on the age of the individual, as well as sex and CAD status at the time of vessel isolation.

### Immunohistochemistry and Immunofluorescence

Microvessels from females <40 and ≥40 yr of age as well as biological males were fixed in 10% zinc formalin for 24 to 72 h. The tissue was then processed, embedded in paraffin, and cross-sectioned into 4-µm slices using the HMS355 microtome. All samples were deparaffinized (Leica Dewax AR9222) and were antigen retrieved in citrate buffer (Leica Epitope Retrieval solution, AR9661). They were then incubated with the following primary antibodies (ER-α Thermo Fisher, MA5-14501 1:50 overnight, PA1-311 1:50 overnight, Cell Signaling 13258S 1:50 overnight; ER-β Thermo Fisher, MA5-13304 1:50 O/N; GPER Thermo Fisher PA5-77396 1:100 overnight). Vessels were costained with CD31 (goat-hosted R&D systems, AF3628 1:100). Samples were then incubated with secondary antibodies (Donkey anti-Goat 488 Invitrogen, A110055; Donkey anti-Rabbit Cy3 Jackson Immuno, 711-166-152) or no-antibody reagent control (negative control). Immunohistochemistry was used to identify ER-α (estrogen receptor alpha Thermo Fisher MA5-14501 1:50 overnight) and detected using the Leica Refine Kit (DS9800). Both immunohistochemistry and immunofluorescence protocols were developed with a Leica Bond Rx immunostaining platform. All slides were DAPI (Sigma, D8417) counterstained and coverslipped (Invitrogen, P36930) and imaged using the Keyence fluorescent microscope at ×20. “Red” signal for each receptor was optimized based on the brightest image, and all slides were then imaged with the same settings (1/60 s exposure with +6-dB gain). DAPI and CD31 exposure times were optimized for each vessel to allow for the identification of the endothelial area based on CD31 staining per vessel. The mean intensity of red estrogen receptor signal within the identified endothelial area was quantified on ImageJ and normalized to the area. For visualization purposes only, a magnified section of each arteriole with a 40% increase in both the brightness and contrast has been included in the top right corner of representative images in Fig. 3.

### Statistical Analysis

Flow response was compared using two-way repeated-measures ANOVA with pressure gradient and treatment as parameters. l-NAME- and catalase-treated groups were each compared with control. Differences between means ± SE with treatment effects are reported in results. One-way ANOVA with Fisher’s LSD test was used for comparing means of maximal dilation to papaverine for various treatments, as well as estrogen receptor expression across groups. Patient demographics were compared using χ^2^, a two-tailed *t* test, or one-way ANOVA. Statistical significance was defined as *P* < 0.05. All analyses were performed using GraphPad Prism, v. 9.1.2.

## RESULTS

Tissue samples from a total of 152 patients were included in the study. Table 1 details the patient demographics for non-CAD males, non-CAD females (< and ≥40 yr of age), and males and females formally diagnosed with CAD via angiographic evidence during cardiac catheterization.

**Table 1.** Patient demographics

	Non-CAD				CAD		
Sample Characteristics (*n* = 152)	Male	Females < 40 yr	Females ≥40 yr	*P* value	Female	Male	*P* value
*n*	22	35	52		10	27	
Age	53.1 ± 10.8	35.5 ± 3.1	54.0 ± 10.6	**<0.001** 0.714*/**<0.001‡**	62.8 ± 8.31	67.0 ± 7.4	0.148
BMI	33.7 ± 9.2	29.2 ± 7.2	29.4 ± 7.0	0.116 0.030*/0.072‡	32.0 ± 10.9	30.5 ± 4.4	0.592
Race, *n*, %	1 (4.5)	2 (6.1)	0	0.419	0	0	**0.006**
Asian	0	4 (12.1)	5 (9.6)		4 (40)	1 (3.7)	
African American	20 (90.9)	28 (78.8)	46		6 (60)	24 (88.9)	
Caucasian	0	1 (3.0)	(88.5)		0	0	
Hispanic	1 (4.5)	0	1 (1.9)		0	2 (7.4)	
Unknown			0		0		
Hypertension, *n*, %	3 (13.6)	0	6 (11.5)	0.094	6 (60)	16 (59.3)	0.967
Diabetes, *n*, %	1 (4.5)	2 (5.7)	2 (3.8)	0.920	5 (50)	11 (40.7)	0.614
Hyperlipidemia, *n*, %	0	1 (2.9)	3 (5.7)	0.460	4 (40)	15 (55.6)	0.401
Congestive heart failure, *n*, %	0	0	0		2 (20)	3 (11.1)	0.482
Smoker, *n*, %	2 (9.1)	1 (2.9)	0	0.092	4 (40)	3 (11.1)	**0.046**

Values are means ± SD or *n* (%); *n*, number of subjects. One-way ANOVA (non-CAD, *top*, *P* value) or two-tailed independent samples *t* test [male vs. female <40 yr (*) or ≥40 yr (‡); CAD males vs. females] was used for comparing age and BMI between groups. Pearson’s χ^2^ test was used for all remaining categorical variables. Boldface indicates significance.

**Figure 1.**
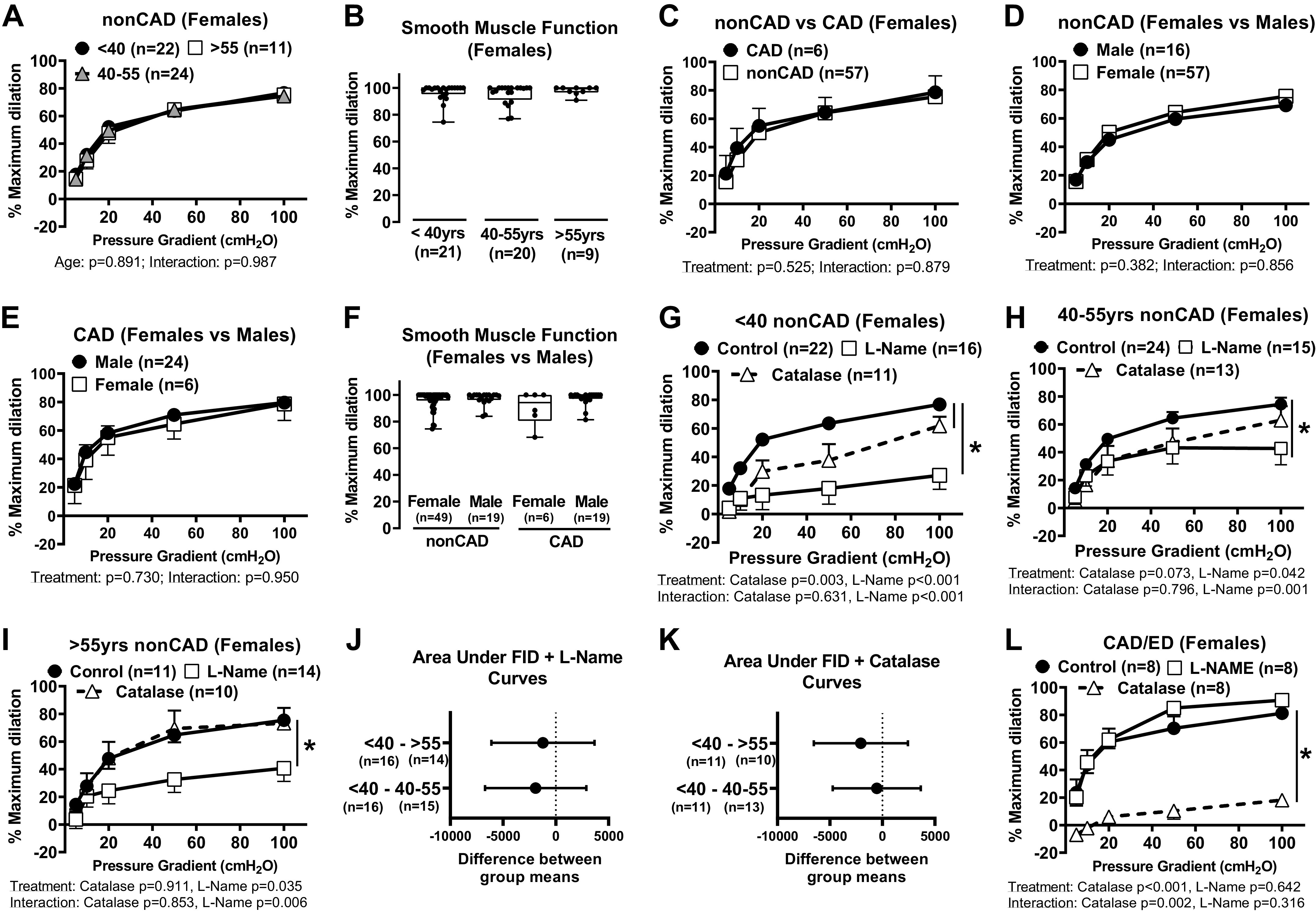
Baseline microvascular function over time and with preexisting disease in untreated arterioles from females and males. *A*: response to flow in arterioles from females <40 (*n* = 22), 40–55 (*n* = 24), and >55 (*n* = 11) yr of age. *B*: maximal dilation to 100 µM papaverine in arterioles from females <40 (*n* = 21), 40–55 (*n* = 20), and >55 (*n* = 9) yr of age (*P* = 0.738). *C*: flow response curves in arterioles from non-CAD (*n* = 57; 30–78 yr) and CAD (*n* = 6; 53–79 yr of age) females. *D*: FID in arterioles from non-CAD males (*n* = 24; 35–74 yr of age) and females (*n* = 57; 30–78 yr). *E*: response to flow in arterioles from females (*n* = 6; 53–79 yr) and males with CAD (49–78 yr). *F*: sex differences in maximal dilation to 100 µM papaverine in arterioles from non-CAD (male, *n* = 49; female, *n* = 19; *P* = 0.890) vs. CAD patients (female, *n* = 6; male, *n* = 19; *P* = 0.078). Response to flow in arterioles from non-CAD females <40 (*n* = 22; *G*), 40–55 (*n* = 24; *H*), and >55 (*n* = 11; *I*) yr of age, with or without *N*^ω^-nitro-l-arginine methyl ester (l-NAME; 100 µM, 30 min) (<40 yr, *n* = 16; 40–55 yr, *n* = 15; >55 yr, *n* = 14) or PEG-catalase (<40 yr, *n* = 11; 40–55 yr, *n* = 13; >55 yr, *n* = 10). Comparison of area under the l-NAME (*J*) and PEG-catalase (*K*) curves for women across age groups. *L*: response to flow in arterioles from females with CAD with or without l-NAME or PEG-catalase (*n* = 8 each). Effect of pressure gradient alone was <0.05 in all groups. *P < 0.05: two-way ANOVA treatment effects (*A*, *C–E*, *G*–*I*, and *L*) and one-way ANOVA with Fisher’s least significant difference (LSD) test (*B* and *F*). CAD, coronary artery disease; FID, flow-induced dilation.

Previous studies indicate that FID remains NO mediated as individuals age (10); however, these data were not separated and analyzed for sex differences. Our results show that the magnitude of dilation in response to flow is maintained (Fig. 1*A*) in untreated arterioles from adult females regardless of age and presumed menopausal status (<40 yr, 40–55 yr, >55 yr for presumably pre-, peri-, and postmenopause, respectively). Smooth muscle function (percent dilation to papaverine) was also similar between these groups (Fig. 1*B*). A reduction in vasodilatory capacity was not observed in arterioles from female patients diagnosed with CAD (Fig. 1*C*). Likewise, no significant differences in FID were found when comparing males versus females from both non-CAD and CAD (Fig. 1, *D* and *E*, respectively) patients, as well as no sex differences in the response to papaverine (Fig. 1*F*). l-NAME treatment significantly reduced FID in arterioles from non-CAD females regardless of age (Fig. 1, *G*–I), and the area under the curve of l-NAME-treated vessels does not vary across age groups (Fig. 1*J*). This suggests that FID remains NO mediated regardless of menopausal status. Although catalase treatment partially reduced FID in arterioles from females < 40 yr of age, maximal dilation to flow was maintained across all groups, and area under the curve of catalase-treated vessels did not vary across groups. This suggests that H_2_O_2_ is not the primary vasoactive mediator produced during increased flow (Fig. 1, *G*–I and K). In microvessels from females diagnosed with CAD, FID was significantly reduced in the presence of catalase, confirming that the vasoactive mediator transitions from NO to H_2_O_2_ with disease (Fig. 1*L*).

**Figure 2.**
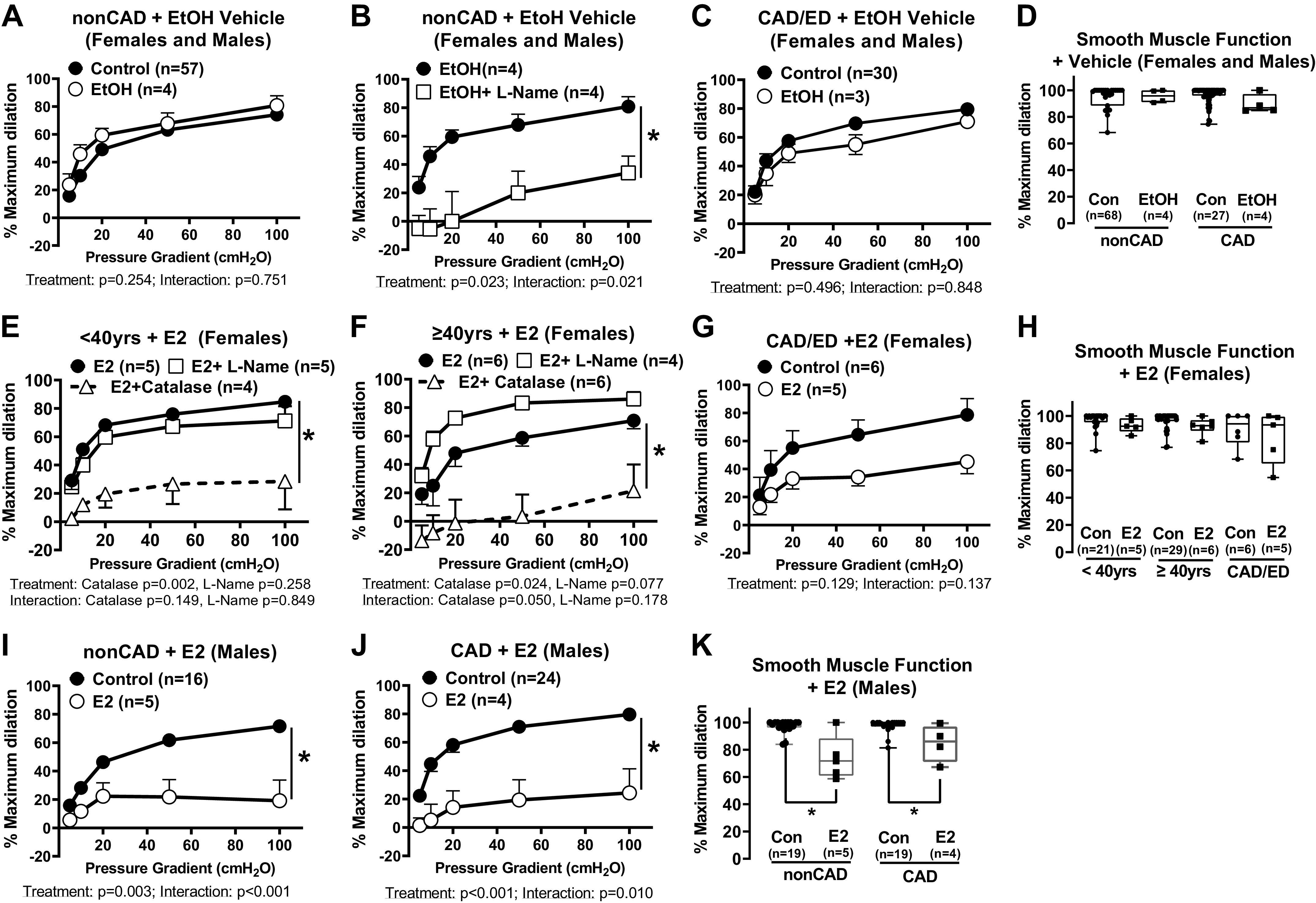
The influence of exogenous estrogen on microvascular function over time and with preexisting disease in females and males. Response to flow in arterioles from non-CAD adults treated with (*n* = 4) or without (*n* = 57) 100 nM EtOH vehicle control (*A*) and in the presence of *N*^ω^-nitro-l-arginine methyl ester (l-NAME; 100 µM, 30 min; *B*) (*n* = 4). *C*: response to flow in arterioles from CAD/ED adults treated (*n* = 3) with or without EtOH (*n* = 30). *D*: maximal dilation to papaverine in arterioles from non-CAD (*n* = 68; EtOH, *n* = 4, *P* = 0.953) and CAD (*n* = 27; EtOH, *n* = 4, *P* = 0.530) patients treated with or without EtOH. Response to flow in arterioles from females <40 (*n* = 5; *E*) and ≥40 (*n* = 6; *F*) yr of age treated with 16–20 h of 17β-estradiol (E_2_), with or without l-NAME (100 µM, 30 min) (< 40 yr, *n* = 5; ≥40 yr, *n* = 4) or PEG-catalase (500 U, 30 min) (< 40 yr, *n* = 4; ≥40 yr, *n* = 6). *G*: response to flow in arterioles from females with CAD (*n* = 6) treated with 16–20 h of E_2_ (*n* = 5). *H*: maximal dilation to 100 µM papaverine in flow in arterioles from females <40 (*n* = 21; E_2_, *n* = 5, *P* = 0.404) and ≥40 (*n* = 29; E_2_, *n* = 6, *P* = 0.322) yr of age and those with CAD (*n* = 6; E_2_, *n* = 5, *P* = 0.258) treated with or without E_2_ (*P* > 0.05 between untreated control < 40 yr, ≥40 yr, and CAD). Flow response curves with (non-CAD *n* = 5; CAD *n* = 4) or without E_2_ (non-CAD *n* = 16; CAD *n* = 24) treatment in arterioles from non-CAD (*I*) and CAD (*J*) males. *K*: maximal dilation to 100 µM papaverine in arterioles from non-CAD (*n* = 19; E_2_, *n* = 5) and CAD (*n* = 19; E_2_, *n* = 4) males treated with or without E_2_. Effect of pressure gradient alone was <0.05 in all groups. *P < 0.05: two-way ANOVA treatment effects (*A*–*C*, *E*–*G*, *I*, and *J*) and one-way ANOVA with Fisher’s least significant difference (LSD) test (*D*, *H*, and *K*). Con and Control denote untreated controls. CAD, coronary artery disease; EtOH, ethanol; ED, endothelial dysfunction.

Based on these results, the remainder of the study grouped females into <40 yr, ≥40 yr (presumably pre- vs. peri-/postmenopausal), and CAD/ED cohorts. Ethanol vehicle alone had no effect on FID or response to papaverine in males and females regardless of age or CAD status (Fig. 2, *A*–D). Dilation to flow was maintained following exposure to exogenous estrogen in non-CAD females [E_2_; 100 nM, 16–20 h; control vs. E_2_ difference between means (DBM); <40 yr females 13.55 ± 7.71%, *P* = 0.091; ≥40 yr females 2.12 ± 6.95%, *P* = 0.762]. Average FID was significantly reduced in the presence of PEG-catalase as opposed to l-NAME in both female age groups compared with E_2_ alone (Fig. 2, *E* and *F*, DBM E_2_ alone vs. E_2_ + PEG-catalase; <40-yr females, 44.04 ± 8.96%, *P* = 0.002; ≥40-yr females 43.96 ± 16.48%, *P* = 0.024). A trend toward impaired dilation was observed in microvessels from females with CAD/ED (Fig. 2*G*) following estrogen treatment compared with untreated arterioles (DBM of untreated vs. E_2_, 22.28 ± 13.32%, *P* = 0.129) and the primary mediator of the remaining dilation was H_2_O_2_ (data not shown). The ability to dilate in response to papaverine was maintained in vessels from females regardless of age or disease status (Fig. 2*H*). Interestingly, unlike female patients, microvessels from males displayed a significant reduction in the magnitude of FID following treatment with exogenous E_2_ (Fig. 2*I*, DBM of untreated vs. E_2_, 27.85 ± 8.45%, *P* = 0.0031). A similar effect was observed in arterioles from male patients diagnosed with CAD (DBM of untreated vs. E_2_, 42.25 ± 10.71%, *P* < 0.001, Fig. 2*J*). A reduction in the response to papaverine following E_2_ treatment was also observed in arterioles from both non-CAD and CAD males (DBM of maximal dilation: non-CAD males, + E_2_, 23.00 ± 3.8%, *P* < 0.001; and CAD/ED males + E_2_, 12.63 ± 4.17%, *P* = 0.004, Fig. 2*K*). These results indicate that arterioles from non-CAD females can compensate and maintain dilation to flow, whereas microvessels from males exhibit both endothelial and smooth muscle dysfunction (lack of response to papaverine) following exposure to E_2_.

**Figure 3.**
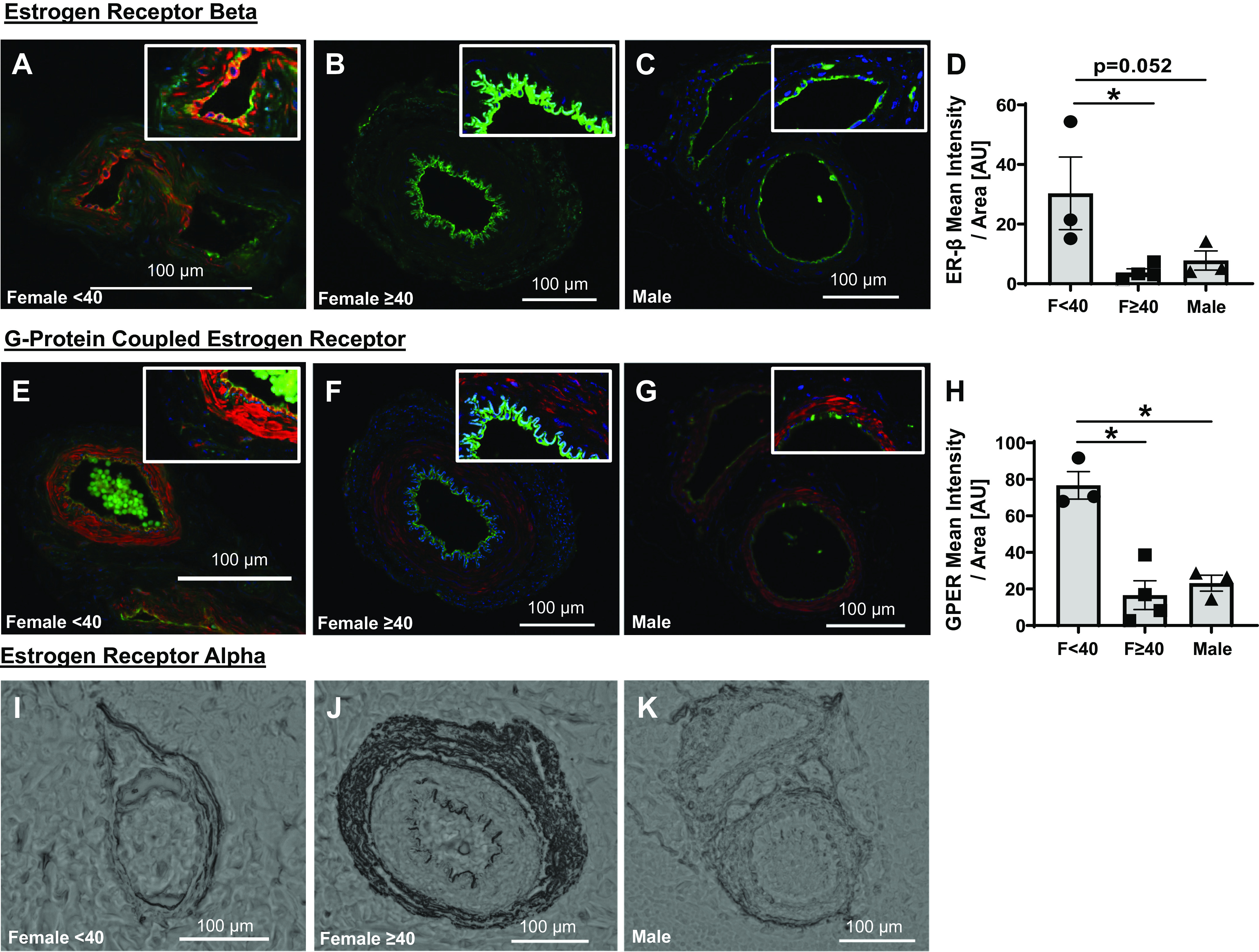
Microvascular endothelial expression of estrogen receptors. Estrogen receptor-β (red, ER-β, Thermo Fisher, MA5-13304; green, CD31, R&D systems, AF3628; blue, DAPI) expression in untreated arterioles from females <40 yr (*n* = 3; *A*) and ≥40 yr (*n* = 4; *B*), and biological males (*n* = 3; *C*). *D*: mean intensity of ER-β normalized to endothelial area identified using CD31 staining. G protein-coupled estrogen receptor (red; Thermo Fisher, PA5-77396) costained with CD31 (green; R&D systems, AF3628) and DAPI (blue) in arterioles from females <40 (*n* = 3; *E*) and ≥40 (*n* = 4; *F*) yr of age, as well as biological males (*n* = 3; *G*). *H*: mean intensity of GPER-signal normalized to endothelial area identified using CD31 staining. *Top*, *right*: for visualization purposes only, magnified sections of each arteriole with a 40% increase in both the brightness and contrast. Representative immunohistochemical staining for estrogen receptor-α (Thermo Fisher, MA5-14501) in females <40 (*I*) and ≥40 (*J*) yr of age, as well as biological males (*K*). *P < 0.05: one-way ANOVA with Fisher’s least significant difference (LSD) test (*D* and *H*). GPER, G protein-coupled estrogen receptor; ER-β, estrogen receptor-β.

Figure 3, *A*–H shows that ER-β and GPER expression within the microvascular endothelium is greater in arterioles from non-CAD females <40 yr of age compared with females ≥ 40 yr of age, as well as adult males. ER-α expression within the endothelium using immunofluorescence staining was undetectable with three different ER-α antibodies that were verified on positive control tissue (e.g., breast/uterus, data not shown). Expression of ER-α was only attainable using immunohistochemistry as this technique provided much-needed signal amplification. As shown in Fig. 3, *I* and *J*, ER-α was identified in the adventitia of the microvessels, whereas little to no staining was observed in the endothelium.

## DISCUSSION

The novel findings of this study are sixfold: *1*) females maintain NO-mediated FID throughout their life span, and maximal dilation to flow is unchanged despite being diagnosed with CAD; *2*) there are no sex differences in the vasodilatory capacity to flow in arterioles from non-CAD and CAD adults; *3*) exposure to an elevated dose of estrogen results in a switch from NO to H_2_O_2_-mediated endothelium-dependent dilation in arterioles from non-CAD females regardless of age; *4*) following exposure to estrogen, a significant reduction in dilation to flow is observed in arterioles from biological males regardless of disease status; *5*) unlike in arterioles from females, there is impairment of endothelial-independent dilation in vessels from biological males following treatment with estrogen; and *6*) there appears to be lower endothelial expression of ER-β and GPER in microvessels from females ≥ 40 yr and males compared with females < 40 yr, whereas ER-α was mainly observed within the adventitia with very little identified in the endothelium.

It has been reported that compared with small arteries from premenopausal women, vessels (∼220 µm) from postmenopausal women exhibit reduced FID, an effect that was restored with estrogen (3 h, 100 nM) (12). Similarly, brachial artery dilation to flow progressively decreases during menopause, and is nearly 50% lower in postmenopausal compared with premenopausal females, an effect restored with estrogen supplementation (13). Although we did not stratify patients based on menopausal status, our data suggest that arterioles from non-CAD females over age 55 yr, who are presumably postmenopausal, continue to dilate maximally to flow in a NO-dependent manner. This highlights a notable difference in microvascular versus small and conduit artery function in females during low estrogen states.

In human endothelial cells, exposure up to 1 μM E_2_ can upregulate eNOS protein levels (14); however, E_2_ in the nanomolar ranges is considered physiological. In the current study, we examined the effect of 100 nM of E_2_, a dose that has been shown to generate NO (2, 3) and improve endothelial function in preclinical models (15). The rationale for using the same dose of estrogen on arterioles from biological males stems from the fact that the goal of gender-affirming therapy is to achieve similar plasma levels of estrogen as compared with cis-females (16). The results presented here suggest that this particular dose of estrogen, despite evidence that it promotes NO signaling in preclinical studies, causes microvascular endothelial dysfunction in microvessels from females diagnosed with and without CAD. Furthermore, there is a significant reduction in both endothelial and smooth muscle function in vessels from biological males.

Potential mechanisms that link estrogen exposure to microvascular dysfunction represent a critical knowledge gap in our understanding of how hormone treatment may contribute to future CVD risk. All three major estrogen receptor isoforms are expressed in large vessel endothelium. Of the three receptors, activation of ER-α is most commonly linked to NO production and vasculoprotection (17), whereas ER-β expression positively correlates with atherosclerotic plaque area in males (18). In fact, some studies have shown that a higher ER-β:ER-α ratio is associated with a prooxidative response to estrogen (19). Given the predominant expression of ER-β in the human microvascular endothelium, as well as the undetectable levels of ER-α, it is possible that estrogen promotes a more oxidative environment in the microvascular endothelium. Also important to note, estrogen concentrations fluctuate in women not undergoing hormone therapy possibly allowing time for recovery. It is also possible that bursts of high estrogen-induced oxidative stress may be beneficial by activating protective pathways. If this is indeed the case, continuous exposure through hormone therapy may not allow for recovery and result in high levels of oxidative stress and vascular damage.

Our study has some limitations. The included subjects are demographically reflective of the surgical population at our institution. For instance, males who undergo surgery that result in discarded samples are often older than females, and as such the number of samples from younger males is limited. This prevented the assessment of age-dependent differences in biological males in this study. It is also not common to receive specimens from trans-females who have undergone gender affirmation and have been exposed to chronic high-dose estrogen; as such, arterioles from biological males were exposed to estrogen in this study as a surrogate for assessing the potential effects of estrogen during gender affirmation among trans-females. Similarly, females diagnosed with CAD also represent a small population of patients which limits the ability to analyze effects based on race and ethnicity. A major limitation is the lack of information regarding menopausal status or hormone use. Furthermore, a variety of progesterones and androgen inhibitors are often clinically combined with estrogen for cis- and trans-females, respectively. Hormone therapy also uses synthetic estrogens, which may differ in signaling compared with E_2_. Future work is needed to validate these initial results in a population with known menopausal status and assess the influence of various clinical hormone therapies on microvascular function. Nonetheless, our methodologies allowed for the evaluation of the direct effect of 17β-estradiol on human microvascular function and for the first time revealed the potentially detrimental sex-specific effects of chronic estrogen in adult males and females regardless of age and disease status.

## DATA AVAILABILITY

Data will be made available upon reasonable request.

## GRANTS

This work is supported by National Heart, Lung, and Blood Institute Grants K08 HL141562 (to J.K.F.) and K08 HL141562-04S1 (to J.K.F.) and American Heart Association Predoctoral Fellowship 909315 (to G.S.K.).

## DISCLOSURES

No conflicts of interest, financial or otherwise, are declared by the authors.

## AUTHOR CONTRIBUTIONS

G.S.K., M.E.S., and J.K.F. conceived and designed research; G.S.K, B.K., H.B.-M., M.Y., E.L.D., and M.E.S. performed experiments; G.S.K. and J.K.F. analyzed data; G.S.K., B.K., H.B.-M., M.E.W., and J.K.F. interpreted results of experiments; G.S.K. prepared figures; G.S.K. and J.K.F. drafted manuscript; G.S.K., B.K., H.B.-M., M.Y., E.L.D., M.E.S., M.E.W., and J.K.F. edited and revised manuscript; G.S.K., B.K., H.B.-M., M.Y., E.L.D., M.E.S., M.E.W., and J.K.F. approved final version of manuscript.
